# Seasonal malaria vector and transmission dynamics in western Burkina Faso

**DOI:** 10.1186/s12936-019-2747-5

**Published:** 2019-04-02

**Authors:** Patric Stephane Epopa, Catherine Matilda Collins, Ace North, Abdoul Azize Millogo, Mark Quentin Benedict, Frederic Tripet, Abdoulaye Diabate

**Affiliations:** 1 Institut de Recherche en Sciences de la santé/Centre Muraz, Bobo-Dioulasso, Burkina Faso; 2 Centre for Environmental Policy, Imperial College London, London, UK; 3 Department of Zoology, University of Oxford, Oxford, UK; 4 Institut des Sciences des Sociétés, Ouagadougou, Burkina Faso; 5 Centers for Disease Control and Prevention (CDC), Atlanta, USA; 6 Centre for Applied Entomology and Parasitology, School of Life Sciences, Keele University, Staffordshire, UK

**Keywords:** Vector control, Genetic control, Seasonal dynamic, *Anopheles gambiae* s.l., Entomological Inoculation Rate

## Abstract

**Background:**

In the context of widespread mosquito resistance to currently available pesticides, novel, precise genetic vector control methods aimed at population suppression or trait replacement are a potentially powerful approach that could complement existing malaria elimination interventions. Such methods require knowledge of vector population composition, dynamics, behaviour and role in transmission. Here were characterized these parameters in three representative villages, Bana, Pala and Souroukoudingan, of the Sudano-Sahelian belt of Burkina Faso, a region where bed net campaigns have recently intensified.

**Methods:**

From July 2012 to November 2015, adult mosquitoes were collected monthly using pyrethroid spray catches (PSC) and human landing catches (HLC) in each village. Larval habitat prospections assessed breeding sites abundance at each site. Mosquitoes collected by PSC were identified morphologically, and then by molecular technique to species where required, to reveal the seasonal dynamics of local vectors. Monthly entomological inoculation rates (EIR) that reflect malaria transmission dynamics were estimated by combining the HLC data with mosquito sporozoite infection rates (SIR) identified through ELISA-CSP. Finally, population and EIR fluctuations were fit to locally-collected rainfall data to highlight the strong seasonal determinants of mosquito abundance and malaria transmission in this region.

**Results:**

The principal malaria vectors found were in the *Anopheles gambiae* complex. Mosquito abundance peaked during the rainy season, but there was variation in vector species composition between villages. Mean survey HLC and SIR were similar across villages and ranged from 18 to 48 mosquitoes/person/night and from 3.1 to 6.6% prevalence. The resulting monthly EIRs were extremely high during the rainy season (0.91–2.35 infectious bites/person/day) but decreased substantially in the dry season (0.03–0.22). Vector and malaria transmission dynamics generally tracked seasonal rainfall variations, and the highest mosquito abundances and EIRs occurred in the rainy season. However, despite low residual mosquito populations, mosquitoes infected with malaria parasites remained present in the dry season.

**Conclusion:**

These results highlight the important vector control challenge facing countries with high EIR despite the recent campaigns of bed net distribution. As demonstrated in these villages, malaria transmission is sustained for large parts of the year by a very high vector abundance and high sporozoite prevalence, resulting in seasonal patterns of hyper and hypo-endemicity. There is, therefore, an urgent need for additional vector control tools that can target endo and exophillic mosquito populations.

**Electronic supplementary material:**

The online version of this article (10.1186/s12936-019-2747-5) contains supplementary material, which is available to authorized users.

## Background

In spite of important and diverse efforts towards control, malaria remains a challenge for the global community and for sub-Saharan countries in particular. The efficacy of current control strategies has stagnated in recent years and, more worryingly, for the first time in a decade, malaria incidence is increasing [1–3]. A number of factors may contribute to this including inadequate financing [2, 4], gaps in control management [5], parasite resistance to drugs [6, 7], and vector resistance to the insecticides used on insecticide-treated bed nets (ITNs) and for indoor residual spraying (IRS) [8–10]. The development of new tools or approaches and the improvement of integrated control strategies are some of the proposed solutions to this resurgence. Novel, genetic control approaches using genetically-modified malaria vectors to achieve population suppression or trait replacement [11, 12] are considered some of the most promising new approaches in an integrated malaria control strategy [13, 14].

West Africa, where high malaria transmission is expected to persist even under optimistic malaria control funding scenarios, might benefit substantially from the development of these novel technologies [15]. Burkina Faso, is one such West African country where the impact of current vector control tools promoted by the Roll-Back Malaria (RBM) initiative led by the World Health Organization (WHO), such as ITNs, IRS and improved malaria diagnostics and treatments, is not expected to bring the country close to elimination [1, 15, 16]. In recent years (2010, 2013 and 2016), wide-scale distribution of long-lasting insecticidal nets (LLINs) has been intensified resulting in coverage ranging from 90 to 97% by 2016 [17]. Although improved diagnosis and reporting structures contribute somewhat to this, the almost doubling of reported malaria cases between 2010 and 2016, rising from 5.7 to 9.8 M [17, 18], suggests that new vector control tools are needed.

One of the first steps towards implementation of any genetic vector control tool consists of gathering key baseline field information: identifying the different vector species present at field sites and evaluating their relative contribution to seasonal patterns of malaria transmission. This understanding is crucial for the planning of mosquito release strategies with optimal health impacts and for demonstrating their effect. The vector species composition patterns identified can match three distinct scenarios: in the first, one mosquito species is strongly dominant and is consequently the target for focussed intervention [19]; in the second case, a dominant species co-occurs consistently with one or more secondary vector species; the third case is that of a less-dominated community in which two or more vectors are more equal in abundance. The second and third scenarios may require more complex release strategies that could include the simultaneous or sequential release of genetically-modified mosquitoes of different taxa.

In western Burkina Faso, members of the *Anopheles gambiae* complex are the dominant malaria vectors [20, 21]. In these Sudano-Sahelian areas, populations of *Anopheles coluzzii*, *An. gambiae* and *Anopheles arabiensis* occur in varying proportions between sites, with large seasonal changes in abundance and a great reduction in numbers, or possibly even complete disappearance, recorded during the dry season [22, 23]. The specifics of an intervention using genetic control tools aiming for vector replacement or suppression [24] would thus depend on the population dynamics of each species and on associated patterns of disease transmission.

The level of malaria transmission can be estimated from many parameters which can be entomological [25], parasitological [26, 27], clinical [27, 28] or immunological [29, 30]. The most common entomological method used is the estimation of the entomological inoculation rate (EIR) by combining an estimated number of mosquito bites per human per day (HBR) [31], which itself is calculated from human landing catch (HLC) data, with information on the prevalence of *Plasmodium falciparum* infections in the salivary glands of mosquitoes, or sporozoite infection rates (SIR). Thus, this estimate of EIR indicates a theoretical maximum number of people potentially infected with *P. falciparum* during a given period (day, month or year) in a given place. The local and seasonal EIR estimates can then be used to inform and focus malaria control strategies.

Baseline entomological collections necessary for informing future strategies and for assessing their potential in reducing malaria transmission were established in three villages typical of Western Burkina Faso. The vectors responsible for malaria transmission were identified and their seasonal abundance was determined using both larval site surveys and pyrethroid spray catches (PSC) indoors. Malaria transmission dynamics were globally estimated by calculating the EIR from HLC and SIR data without digging into specific species contribution to this. Finally, local rainfall data were measured using a permanent weather station at each site. The results highlight the challenges resulting from very high vector densities characterizing West African Sudano-Sahelian regions during the rainy season.

## Methods

For over 3 consecutive years (July 2012–November 2015), monthly mosquito field collections to track vector composition, abundance and related malaria transmission, took place in three villages in Burkina Faso’s Sudano-Sahelian region near Bobo-Dioulasso. Three collection methods were used each time: larval site surveys, indoor pyrethroid spray catches (PSC) and human landing catches (HLC).

### Study sites

**Fig. 1:**
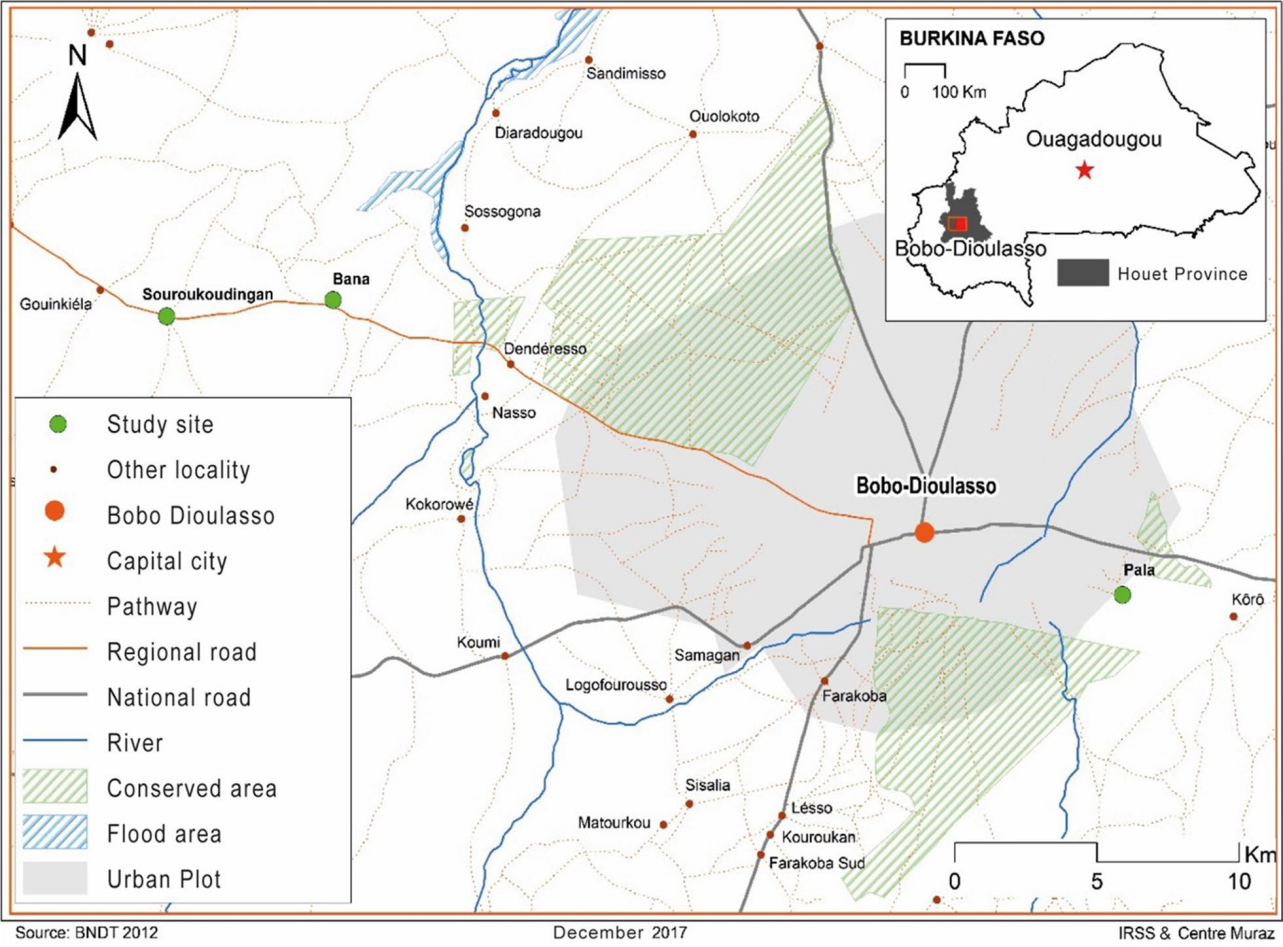
Location of the village study sites in the Houet Province of western Burkina Faso

The survey was conducted in the villages of Bana, Pala and Souroukoudingan (Fig. 1, Table 1), all located in the western Burkina Faso humid savannah zone. This region is characterized by two seasonal extremes: a wet season from June to September and a dry season from November to April, with October and May being transition months. The mean annual rainfall regionally is about 800 mm (maximum in September, minimum in January) with a mean temperature of about 27 °C (22 °C monthly mean minimum and 32 °C monthly mean maximum). The annual mean relative humidity is 59%, but varies substantially with rainfall and surface water (31% monthly mean minimum and 87% monthly mean maximum).

**Table 1:** Description of the study villages

Study village	Bana	Pala	Souroukoudingan
Population size (2014)	750	2400	830
Number of compounds	130	245	103
Location (longitude)	− 4.477778	− 4.423333	− 4.5336389
Location (latitude)	11.236667	11.150556	11.235278
Distance to Bobo-Dioulasso (km)	23	6	29
Context	Rural	Peri-urban	Rural
Water relations	Semi-permanent river, impermanent seasonal pools	Semi-permanent rivers, permanent pools	Seasonal pools
Economy	Major: trade and arable subsistence Minor: livestock farming	Major: trade and handicraft Minor: arable subsistence and livestock farming	Major: arable subsistence Minor: livestock farming

The sites were selected to represent villages representative of this region and also to be accessible from the research base in Bobo-Dioulasso. Bana has two main inhabited areas, Bana Centre and Bana Market, separated by a small semi-permanent river (usually get dry during the dry season). Bana Centre is the principal agglomeration and includes the village administration and a small health centre. Bana Market is the economic centre of the village and hosts a busy weekly market. The whole village is a loose cluster of about 130 compounds (local census, October 2014**)**. Each compound is a family unit consisting of between two and ten houses, mostly mud-built. The main economic activities in the village are arable subsistence farming and stock farming. Souroukoudingan is about 6 km west of Bana. It is similar to Bana in geographic and socio-demographic characteristics, but is further from a river, has less surface water and is a single cluster of about 103 compounds. The third village, Pala, is a peri-urban village located 6 km south-east of central Bobo-Dioulasso. The village is crossed by two small rivers which usually remain wet throughout the year. Relatively populous in comparison with the other studied villages, Pala has about 245 compounds for about 2400 inhabitants. Here the main activities are increasingly urban-focussed with trading and handicraft and lesser arable subsistence and stock farming (Table 1).

### Larval site surveys

A longitudinal survey of larval habitats was carried-out using classical larval prospection and sampling techniques, to assess the presence of potential breeding sites in the villages throughout the year. One day each month, all potential larval habitats were explored, described morphologically (type and size) and georeferenced using a GPS device (Garmin GPS series GPSMAP^®^62.2.3). All were scouted for the presence of *Anopheles* larvae or pupae identified morphologically at the gender level, using the anopheline larvae morphological identification keys developed by Holstein in 1949 [32]. The finding of at least one larva or pupa was sufficient to record a larval habitat as occupied (effective breeding site); no further quantitative estimates were made.

### Mosquito abundance estimates

From July 2012–November 2015, indoor resting mosquitoes were collected monthly by PSC to estimate the variation in their abundance over time. Twenty compounds were selected per village and sprayed once each month for this study. Ten of these were “fixed” (always sampled) and the others were randomly selected from those remaining in the village. The fixed compounds were spread to represent the geography and extent of the village. For each selected compound in Bana and Souroukoudingan, a single room was sprayed. In Pala, because of particularities in house structure (commonly no door between rooms in a house), all rooms were sprayed in the house and the mean number per room was calculated. The insecticide spray used for PSC was Kaltox Paalga^®^ (Saphyto, 1937 Avenue du général Sangoulé Lamizana, Bobo-Dioulasso, Burkina Faso). This is commercially available and commonly used locally. The collected mosquitoes were identified morphologically in the field using the adult anopheline morphological identification keys developed par Holstein [32] and a field stereomicroscope (Perfex Sciences^®^ Zoom Pro. Reference: S0852Z5 Toulouse, France), counted and preserved in 80% (v/v) ethanol for subsequent species identification by polymerase chain reaction (PCR) analysis.

### Species composition

Molecular analysis of samples collected by PSC were performed by PCR, to identify the species of *Anopheles* vectors found in the villages. This allowed determination of the relative proportion of each species within the *An. gambiae* complex for each village. The PCR technique used was based on the detection of SINE 200× locus [33].

Ninety mosquitoes were selected for PCR from each village’s monthly catch (or all available mosquitoes when there were fewer than 90). The sub-sampling was performed to give a representative selection with each sampled compound contributing in proportion to the total mosquitoes caught that month in the village. Two legs were removed from each sub-sampled mosquito these were placed in PCR reactions using the Gotaq^®^ PCR kit (GoTaq^®^ G2 Flexi DNA Polymerase, reference: M829B, Promega Corporation, 2800 Woods Hollow Road·Madison, WI 53711-5399, USA).

### Seasonal malaria transmission estimates

To estimate the monthly malaria transmission rate in each village, the human landing rate (HLR) of mosquitoes (from HLC) and the mosquito sporozoite infection rate (SIR) were used to calculate the entomological inoculation rate (EIR).

### Human landing rate

Human landing catches (HLC) serve to estimate the human landing rate and thus the number of potential bites per human per day [31]. In each village, four houses were chosen, in such a way to have a representative geographical distribution and during four consecutive nights each month (the same nights in all villages), two collector stations (one indoor and one outdoor) at each house collected all skin landing (pre-biting) mosquitoes from 20:00 to 06:00 the next morning. Collectors (all adult males) were regularly rotated to reduce collector-mediated bias in the results and supervision was provided to ensure collectors stayed awake thus reducing any potential for biting. The mosquitoes collected in each house were stored by collection origin (indoor and outdoor) and in hourly tranches. As with the PSC catch, mosquitoes were identified morphologically and all the *An. gambiae* sensu lato (s.l.) mosquitoes were preserved in 80% (v/v) ethanol for further analysis. The nightly mean landing rate for each village was then calculated.

The study received approval from Institutional Ethics Committee of the IRSS, Centre Muraz and all collectors received appropriate information, were given appropriate supervision and gave their prior informed consent to participate to the study.

### Sporozoite infection rate and entomological inoculation rate

The EIR is the product of the HLR and the SIR [31], where the SIR is defined as the fraction of the HLC *An. gambiae* s.l. that are infectious. This latter was determined by enzyme linked immunosorbent assay of circumsporozoite protein (ELISA-CSP) analysis.

For each village, 60 *An. gambiae* s.l. (or all available mosquitoes when there were fewer than this) were selected each month from the HLC collections. Each house and indoor/outdoor collection point was represented proportionately. The head and thorax of selected mosquitoes were removed and used for the ELISA-CSP analysis. The reagents and protocol used were the ones applied by Wirtz et al. in 1987 [34]. All positive samples were reanalysed twice for confirmation. The monthly SIR was determined as the proportion of infected mosquitoes in the total tested. EIR was then estimated monthly for each village as the product of estimated HLR and its corresponding SIR.

### Local rainfall data

Rainfall data were collected using Onset Hobo weather stations (Hobo RX3000, Onset, Bourne, MA, USA) installed in June 2014 in each village to correspond with local mosquito abundance. For survey dates prior to the installation of the weather stations, data from the same date for all subsequent years in which weather data were available were averaged to provide an informed indication of typical local rainfall during that period of survey.

### Data analysis

Vector abundance, relative proportion of the different species of malaria vectors, human landing rate, sporozoite infection rate and entomological inoculation rate were estimated monthly for each study village. These variables were separately compared from one period to another using either proportion tests or binomial-family generalized linear models (GLMs) with stepwise factor level reduction testing. Analysis of variance (ANOVA) with stepwise deletion testing was used to assess the influence of place, season, collection period (months and years) on the human landing rate. The degrees of freedom presented with F values are those associated with the factor of interest and the error/residual degrees of freedom of the model. Parameter estimates, such as mean values, are presented with their 95% confidence intervals (95% CI).

## Results

### Larval site surveys

During the sampling period, a total of 874, 521, and 1011 potential breeding sites (water locations) were recorded in the villages of Bana, Souroukoudingan, and Pala, respectively. In Bana, rain puddles (69.57%) and tyre tracks (19.57%) which are short duration (< 7 days) larval sites, were the most frequently observed. Some (less than 1%) small quasi-permanent water pools (0.02–0.5 m^2^) were also observed in the surroundings of the village pomp (overflow ponds) and along the river that separate the two main agglomerations of the village (Bana Centre and Bana Market). In Souroukoudingan, the most frequently observed larval sites were tyre tracks (38.2%), retention ponds (23.8%) and brick pits (16.32%) which are short and medium duration (typically > 7 and < 14 days) larval sites. The majority of these were medium to large in size (1–10 m^2^ surface area). In Pala, the majority of larval sites were small water pools (0.02–0.5 m^2^) along the river beds (84.47%) and brick pits (13.25%). These rivers remain wet all through the year (contrary to the river of Bana village which usually dries out during the dry season) and these small water pits were quasi-permanent.

**Fig. 2:**
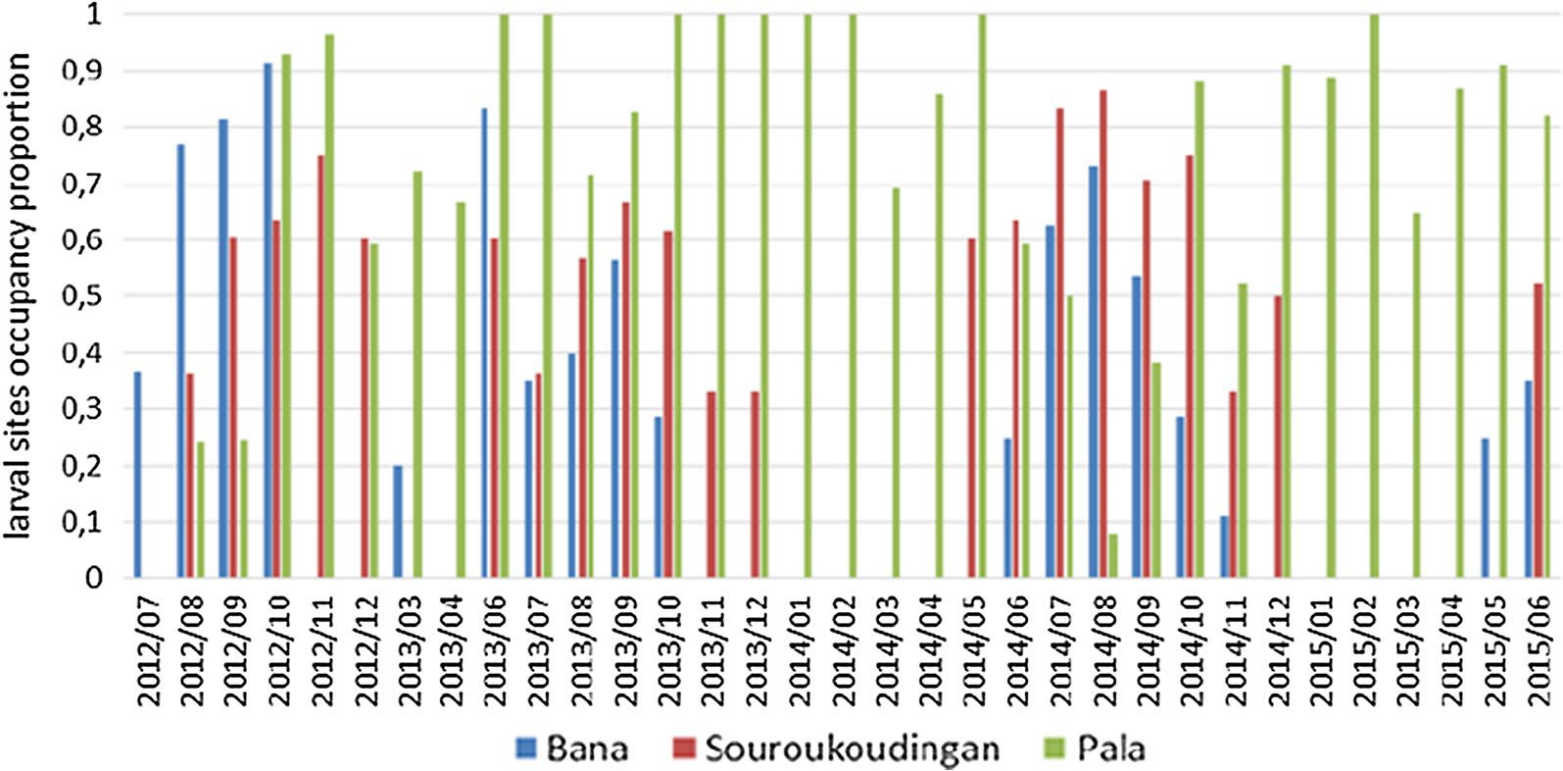
Larval sites occupancy across the period of study. Time-series of larval site occupancy proportion in the village of Bana, Pala and Souroukoudingan across the period of study

The occupation proportion of larval sites by *Anopheles* larvae or pupae varied between villages (*F*_(2, 0)_ = 34.65, *P*  < 0.0001). In Bana 54.5% of all the observed larval sites were occupied, in Souroukoudingan this was similar at 55.3% and in Pala, a higher occupancy at 73.3% was observed. The larval site occupation was also very heterogeneous across the year (Fig. 2), strongly correlated with wet season in the village of Bana and Souroukoudingan (higher occupancy during the wet season and lesser or not at all during the dry season) but not in the village of Pala.

### Seasonal abundance

In all villages, the great majority of *Anopheles* mosquitoes collected by PSC were members of the *An. gambiae* complex (about 99%, see Additional file 1). During the sampling period, a total of 14,509, 8222, and 11,189 *An. gambiae* s.l. mosquitoes were collected by PSC in the villages of Bana, Souroukoudingan and Pala, respectively. In Bana and Souroukoudingan, more *An. gambiae* s.l. mosquitoes were collected per house during the wet season (June to October) than in the dry season (November–May) (Bana: *t* = 4.66, *df* = 32, *P* < 0.0001; Souroukoudingan: *t* = 5.56, *df* = 32, *P*  < 0.0001). In Pala seasonal difference was not identified (*t* = 1.75, *df* = 32, *P *= 0.09).

### Species composition

**Fig. 3:**
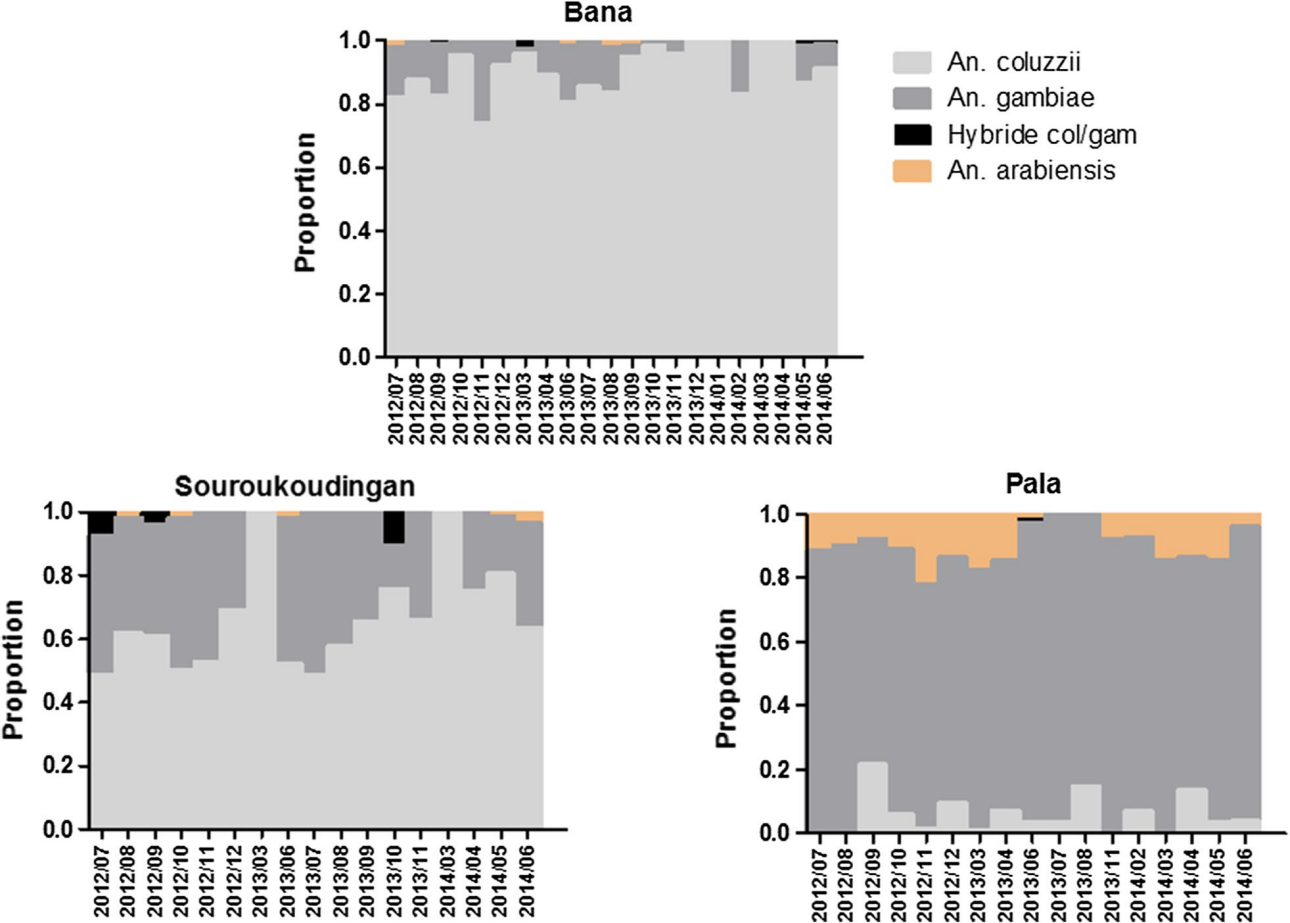
Proportional species composition of the *Anopheles gambiae* s.l. malaria vector population in each village over a two year period. Hybrids between *An. coluzzii* and *An. gambiae* (col/gam) were detected, but occurred rarely

In Bana and Souroukoudingan, *An. coluzzii* and *An. gambiae* were the principal malaria vectors found (Fig. 3). In Bana, *An. coluzzii* dominates the catch throughout the year with a mean of 90.46% (86.95–93.97). Here, *An. gambiae* is present, but minor in comparison at 8.89% (5.46–12.32) of the catch. In Souroukoudingan, *An. gambiae* increases in abundance rising to 31.72% (23.63–39.81) of the catch, but *An. coluzzii* remains dominant at 66.34% (58.15–74.53). Occasional *An. arabiensis* were found (less than 1%), mainly during the dry season. In Pala, three major malaria vectors were observed: *An. gambiae, An. coluzzii* and *An. arabiensis.* The most abundant in proportion was *An. gambiae* at 84.18% (80.32–88.04) of catch, followed by *An. arabiensis* and *An. coluzzii* with 9.98% (6.80–13.16) and 5.76% (2.63–8.90) respectively. Some hybrids of *An. coluzzii* and *An. gambiae* were also found. Except for the village of Souroukoudingan where they reached 1.29% (0.00–2.88), the relative proportions of these hybrids were usually very low. In the village of Bana their proportion was of 0.31% (0.00–0.63) and 0.07% (0.00–0.23) in the village of Pala.

### Seasonal malaria transmission

#### Human landing rate

Human landing catches consisted almost entirely (99%) of mosquitoes in the *An. gambiae* complex seeking blood meals. There was no identified variation in catch numbers between the inside and outside of houses in any of these villages (Bana: *t* = 0.46, *df* = 286, *P* = 0.46; Souroukoudingan: *t* = 0.94, *df* = 286, *P* = 0.35; and Pala: *t* = 1.88, *df* = 286, *P* = 0.06) and this factor was not considered further (see Additional file 2).

**Fig. 4:**
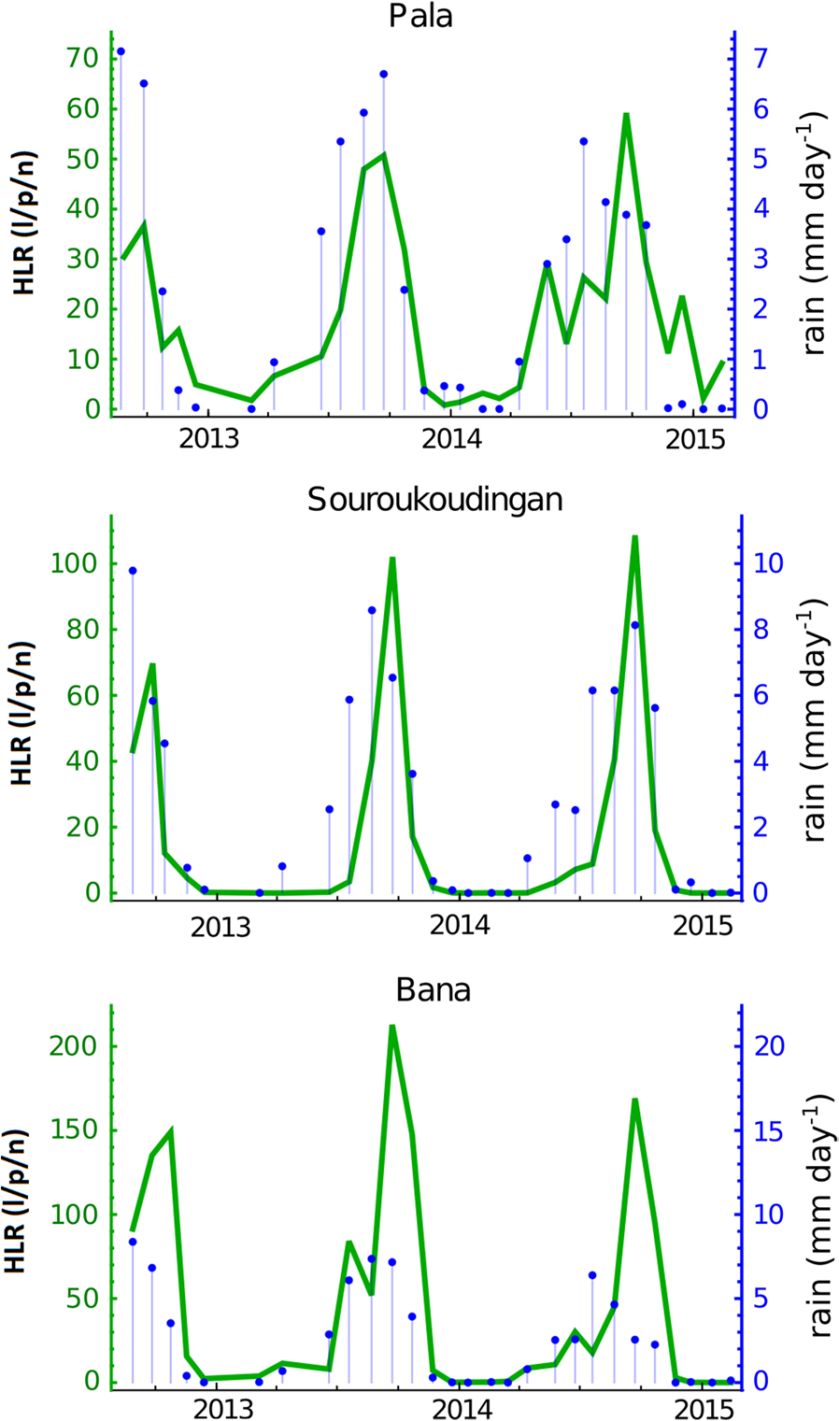
Human landing rate time series and corresponding rainfall. The time-series of human landing rate data (HLR) for each village (green), estimated via data from human landing catches, and rainfall from the week immediately preceding each survey (blue). HLR is expressed in “l/p/n” (number of human landing mosquitoes per person and per night)

The three villages exhibited similar cyclic variation in human landing rate with a peak during the wet season and a decline in the dry season (Fig. 4). There was variation, however, between the villages in the rate itself (*F*_(2, 429)_ = 63.00, *P* < 0.0001): during the wet season, Bana had an average of 92 (79–106) landings per person/night; in Souroukoudingan and Pala this was lower with about 35 (28–43) and 29 (25–32) landings per person/night respectively (Sourououdingan: *t *= 7.74, *df *= 286, *P* < 0.0001; Pala: *t *= 9.58, *df *= 286, *P* < 0.0001). During the dry season, the landing rate falls at all sites (*F*_(2, 429)_ = 39.76, *P* < 0.0001), though in Pala (8 (6–10)) this remains higher than those of the other sites: Bana (4 (3–5), *t *= 3.56, *df *= 286, *P* < 0.001) and Souroukoudingan (1 (0–1), *t *= 8.16, *df *= 286, *P* < 0.001). This pattern mirrors the pattern observed in PSC abundance data. The main factors responsible for the observed variation of the human landing rate was in order of importance the season and the locality.

**Fig. 5:**
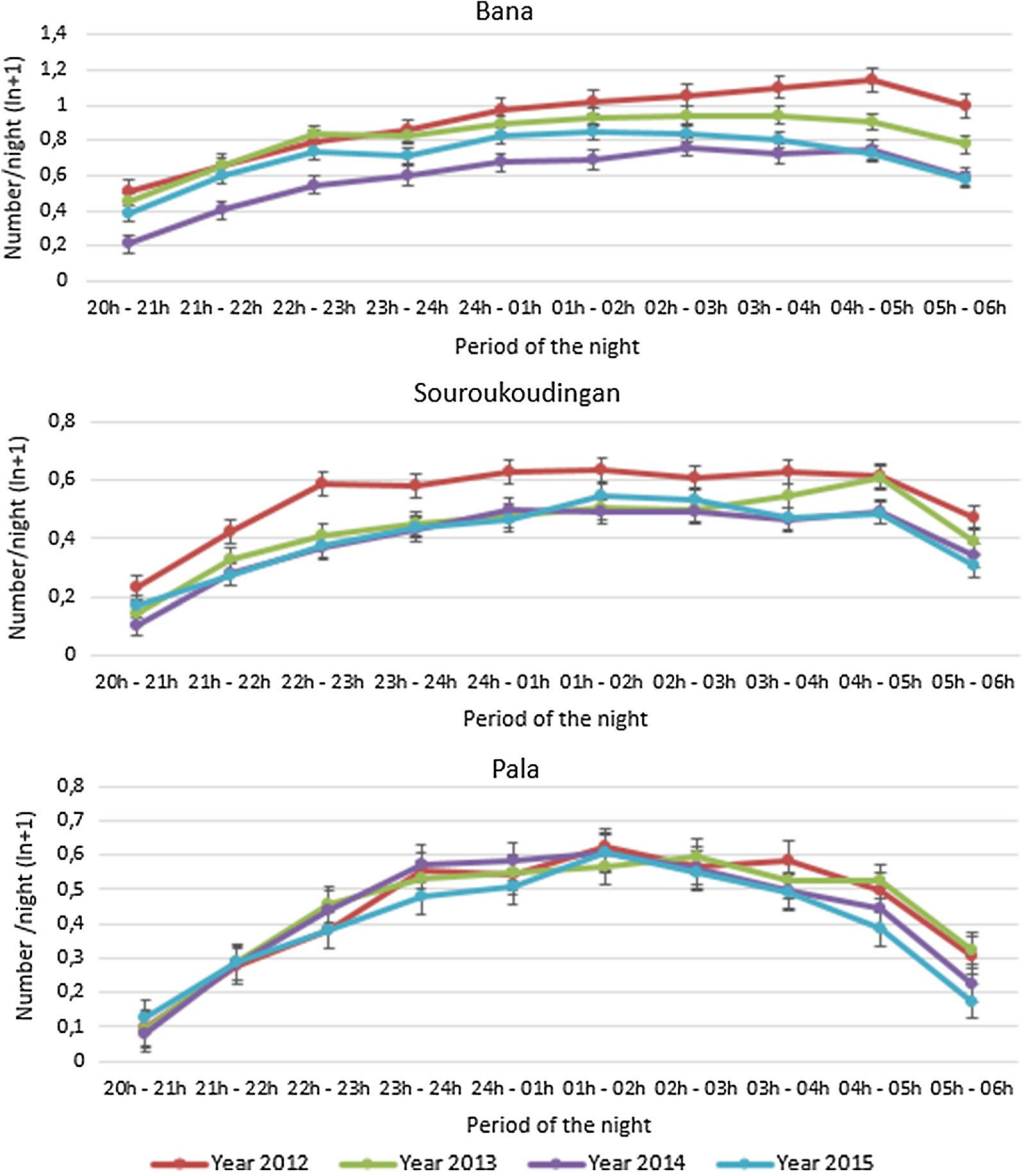
Mosquito human landing pattern at each studied village as a function of time of night. The abundance of skin landing mosquitoes (LnN + 1) per person through the night in hourly tranches

The time of night that landings are observed varies consistently between villages and these very local patterns persist between years (Fig. 5). In Bana, mosquito catch peaks between 03:00 and 05:00, in Souroukoudingan the numbers are spread more evenly through the night and in Pala the catches strongly peak between 23:00 and 04:00.

#### Sporozoite infection rate

The SIR was estimated each month in each village as the proportion of infected (ELISA-CSP positive) mosquitoes from HLC captures (Table 2, Additional file 3). In Bana, the mean SIR was 3.12% (1.89–4.96), in Souroukoudingan it was 6.56% (1.61–11.51) and in Pala, 4.18% (1.86–6.50), there was thus no variation in SIR between villages detected here (*F*_*(2, 76)*_=0.365*, P *=0.695). Similarly, the wet season and dry season values in each village did not indicate seasonal differences (Bana: *t* = 0.870, *df* = 28, *P* = 0.392; Souroukoudingan: *t* = 0.871, *df* = 20, *P* = 0.394 and Pala: *t* = 1.035, *df* = 25, *P* = 0.310). These results suggest that the SIR remained similar from one locality to the other and from one season to the other in the studied sites. No significant variation was detected too in annual values of the SIR from 2012 to 2015 (Bana: *F*_*(3, 26)*_=0.240*, P *=0.867; Souroukoudingan: *F*_*(3, 18)*_ = 1.288*, P * = 0.309), except in the village of Pala (*F*_*(3, 23)*_ = 5.118*, P * = 0.007), which difference was due to the 2012 value of the SIR (annual variation no significant for years after 2012; *F*_*(2, 14)*_ = 1.495*, P * = 0.258).

**Table 2:** Annual monthly mean estimates of the *Plasmodium falciparum* sporozoite infection rate (SIR) in the studied villages

Year	Bana	Pala	Souroukoudingan
2012	0.041 (− 0.006 to 0.088)	0.099 (0.021–0.178)	0.047 (− 0.014 to 0.108)
2013	0.031 (− 0.010 to 0.073)	0.076 (0.003–0.149)	0.125 (− 0.016 to 0.266)
2014	0.033 (− 0.005 to 0.071)	0.017 (− 0.002 to 0.035)	0.027 (− 0.003 to 0.057)
2015^a^	0.013 (− 0.013 to 0.038)	0	0.011 (− 0.125 to 0.146)

Means are presented with their IC (95% confidence interval)

^a^In 2015, only data from January to June (dry season period) were included in the estimates (see Additional file 2 for further details)

#### Entomological inoculation rate

In Bana, the mean overall EIR was 1.078 (0.056–2.101) infectious bites/person/night (about 394 potential infectious bites/person/year); in Souroukoudingan this was 0.416 (0.162–0.669) (about 152 potential infectious bites/person/year) and in Pala, 0.547 (0.177–0.917) (about 200 potential infectious bites/person/year). The EIR is driven by SIR and HLR. Since no significant variations was detected in SIR data between season and localities (villages), the EIR should be largely driven by the HLR and the level of variation in the former drives almost similar conclusions even though no variation between villages was detected here (*F*_(15, 79)_ = 1.78, *P* = 0.053) as compared to HLR. Each village had a higher EIR during the wet season than in the dry (Bana: *t* = 2.403, *df* = 30, *P* = 0.023; Sou.: *t* = 4.514, *df* = 30, *P* ˂ 0.0001; and Pala: *t* = 2.102, *df* = 30, *P* = 0.044). In Bana the EIR wet season mean was of 2.350 (0.038–4.662) potentially infectious bites/person/night and falls 25-fold to a dry season mean of 0.089 (0.000–0.185). Similarly, in Souroukoudingan, EIR estimates decreased from 0.914 (0.437–1.391) to 0.028 (0.000–0.086) from wet to dry season and in Pala this was from 0.957 (0.141–1.773) to 0.228 (0.037–0.418).

**Fig. 6:**
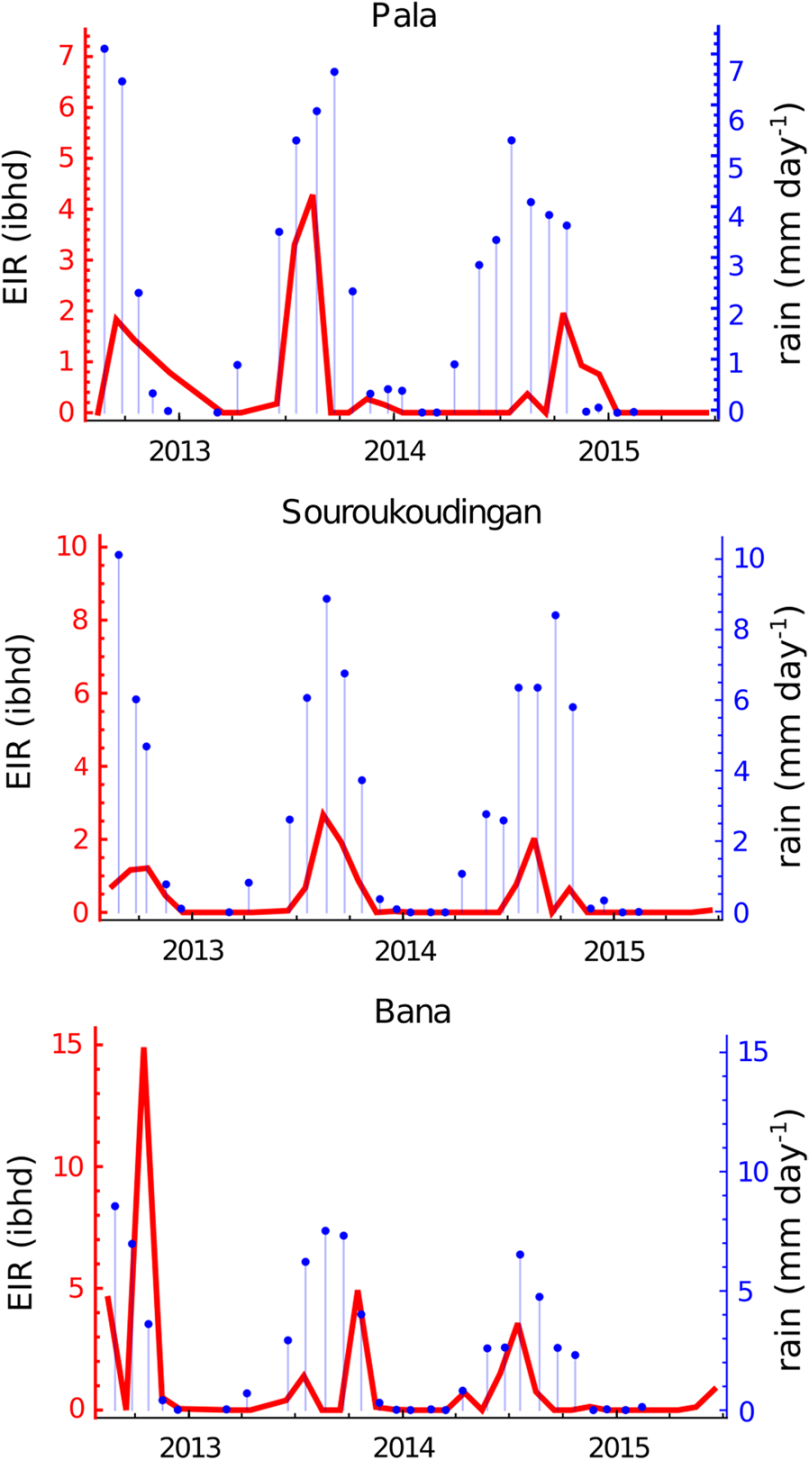
Entomological inoculation rate time series and corresponding rainfall. The time-series of entomological inoculation rate (EIR) data for each village (red) and rainfall from the week immediately preceding each EIR calculation (blue). EIR values are expressed here in “ibhd” (potential number of infectious bites per human and per day)

The strongest influence on the EIR was thus the season (*F*_(1, 89)_ = 25.51, *P*  <  0.001). The highest EIR estimated was in Bana in October 2012 with about 15 potentially infectious bites/person/night, meanwhile Pala and Souroukoudingan reached peak estimated EIR in August 2013 with 4 and 3 potentially infectious bites/person/night respectively (Fig. 6).

## Discussion

The malaria vector dynamics in these three study sites contrast in several ways: Species composition, abundance, larval site occupancy and in SIR and EIR. Although the sites are close to one another within the same province, Houet, there are contrasts in the local ecological conditions that may contribute to this. In Bana and Souroukoudingan, the majority of larval sites were rain puddles and tyre tracks which are ephemeral (short-term) larval sites and highly rain-dependent, though Bana has some longer-term larval habitat availability in a semi-permanent river which persists for longer into the dry season and a quasi-permanent overflow pond at the village pump. In contrast, in Pala the most common larval sites were small water pools located along the beds of the two small rivers that cross the village. These pits are also quasi-permanent and remain wet year-round. Larval site occupancy was higher in Pala than in the two other villages, possibly due to the availability of these small, enduring and numerous potential larval sites.

The water relations in the villages may also influence the species composition of the vector community. The malaria vectors identified in this study were mostly within the *An. gambiae* complex (*An. coluzzii*, *An. gambiae*, and *An. arabiensis*), and other vector species known in Burkina Faso, *Anopheles funestus* [21, 35, 36] and *Anopheles nili* [37], were found occasionally. This is consistent with expectations of this ecological zone as the type of larval sites found in the Sahelian climate do not favour the development of *An. funestus* or *An. nili*, which are usually found where there is running water [20, 38]. There are ecological differences in the larval habitat preferences within the *An. gambiae* complex too. *Anopheles coluzzii* larvae are often associated with larger, more permanent breeding sites while *An. gambiae* larvae are associated with small and short-term breeding sites [39, 40]. In Bana and Souroukoudingan, where *An. coluzzii* dominated the catch, the majority of breeding sites were short-term, though generally of large size (3–10 m^2^). In Pala, where *An. gambiae* dominated the catch, they were mainly quasi-permanent but usually of small size (0.02–0.5 m^2^). This suggests that the size rather than the level of permanency may be more influential on the mosquitoes’ development at larval stage and thus on species composition. In fact, the size of larval habitat is known to have an important influence on the response of *Anopheles* larvae to predation [41]. The contrast in predation response between these species may thus influence larvae colonization of larval habitats [40, 41]. Some other factors such as water turbidity, colour or sunniness could have also contributed up stream to this colonization. Unfortunately these factors were not described in this study.

Mosquito abundance patterns identified in HLC in the three villages agree with those observed by PSC collections: *An. gambiae* s.l. constitutes the principal suite of malaria vectors in the three villages and there is a high correlation between the techniques for estimates of vector densities through the year. Both techniques provide a good index of relative abundance and species composition in a given area and the differences observed between villages, such as the very high wet-season populations in Bana and the residual dry-season populations in Pala were supported by both techniques. Sahelian anopheline populations are strongly seasonal, peaking in the rainy season [22, 23] and all of the study sites strongly conform to this pattern. The presence of a dry-season population in Pala reflects and responds to the presence of enduring larval habitat in this village.

A vector’s biting rate is one of the most important parameters to influence malaria transmission in a given locality [42–44] and the most direct way to estimate this parameter is through human landing catches (HLC). The use of HLC allows for exploration of another aspect of mosquito behaviour that PSC does not give: endophagy *versus* exophagy. Here, no difference in landing rate was observed between indoor and outdoor collections and suggests that in this region, malaria vectors have an opportunistic biting behaviour. Endophagy is usually the expected dominant behaviour in an *An. gambiae* s.l. mosquito population and the degree of exophagy observed here has been recorded previously in South Africa [45, 46]. This may result from a modification in host-seeking behaviour induced by years of vector control using ITNs [47, 48]. Mosquito species of the *An. gambiae* complex are known to be heterogenous in their biting behaviour. Unfortunately, species composition of the HLC collections are not available in this study. This could have given a more precise information about the contribution of each species in the observed general biting behaviour of the mosquitoes caught. However, at least in a context of high dominance of one species, as observed in the villages of Bana and Pala, it could be considered that species composition of PSC collection should not be too much different from the HLC ones.

The landing time data over the 4 years of this study does not indicate a shift in host seeking towards dusk or dawn when people are more mobile and less likely to be protected by their bed nets. Each village has a locally consistent pattern and, in each, most landings were observed between 22:00 and 05:00. Extending the duration of the HLC an hour in either direction would provide the means to detect possible behavioural change towards day biting. In the absence of evidence of a local behavioural shift, the night landing patterns observed here confirms the crucial importance of ITNs as a malaria vector control tool.

An early-biting pattern could potentially explain a high sporozoite infection rate (SIR) and related EIR [47, 48], but this was not seen to be the case at the sites studied during this survey. Another factor which may contribute to residual EIR in the dry season around Pala is a change in host behaviour; with increasing urbanization there is a shift in human activity that leads to increased host availability into the dark hours [49].

The estimates of SIR and related EIR in this study emphasize the magnitude of the remaining malaria burden, despite good local implementation of the national vector control strategy. Although ELISA-CSP is known to overestimate SIR by a factor of about 1.1–1.9 [50], there is discussion surrounding the accuracy and level of inference to be drawn from these traditional calculations of EIR [51, 52], especially with the low sample size used in this study, these values remain surprisingly high in this local context [53]. This region has one of the highest rates of ITN coverage in Burkina Faso with a mean coverage of about 92% and a usage rate over 72% [17, 18].

Three successive ITN (or LLIN) mass distribution campaigns occurred in Burkina Faso in 2010, 2013 and 2016 with excellent results in term of household coverage [18]. The expectation of a significant reduction in EIR via action on its components: landing rate or sporozoite infection rate, or both, has not been observed in the years of this study. This agrees with national patterns of malaria incidence and morbidity which have continuously increased during the same period, rising from about 5.7 M malaria cases in 2010 to about 9.8 M cases in 2016 [17, 18]. No significant increase in EIR was observed during this study which may suggest at least a stabilization of malaria transmission in this region and these findings may indicate a qualified success (though insufficient) of the currently implemented malaria control tools in this region.

Potential reduction in efficacy of current malaria vector control tools is not specific to the areas studied during this survey; an increasing number of malaria endemic countries of diverse mosquito ecology retain high malaria transmission parameters despite a good implementation of ITNs and/or IRS. This is the case in an Ethiopian region where residual transmission is observed in spite of extensive coverage by IRS and LLINs [54]. In a forest village of Cameroon (Central Africa), Ndo et al. [55] observed recently a SIR of 3.87% in *An. gambiae* mosquitoes. In Mali, the African IRS project has described long-term non-effects of IRS and LLINs in a number of areas [56].

Over these last two decades, LLINs and IRS have had an exceptional impact in reducing malaria and other vector-borne disease transmission worldwide [3]. Even now, their contribution to the reduction of malaria incidence and morbidity represents the largest proportion of global impact in malaria control [3]. Unfortunately, a reduction of their efficacy is increasingly observed at different sites. Insecticide resistance in mosquitoes [55, 57] and biting behaviour changes [47, 48] are thought to be the main contributors. All these results sustain the thesis of Shaukat et al. [25] which suggests that current vector control methods cannot ultimately eradicate malaria. So, with an aim to malaria eradication in the Sudano-Sahelian region, it is increasingly urgent to research and develop novel vector control tools particularly designed to suppress its very large and seasonal malaria vector populations. In that context, novel genetic control approaches based on male mosquito release programmes may prove a crucial additional tool in the toolbox of future integrated malaria control programmes.

## Conclusion

This study has allowed a better understanding of seasonal dynamics of malaria vectors in the villages studied and the main factors that influence these. The vectors found in these three villages belong mostly to the *An. gambiae* complex (*An. coluzzii*, *An. gambiae*, *An. arabiensis*), but with variable relative proportion from one locality to another. Local geographic and hydrographic differences could explain, at least partially, the differences in vector abundance from one locality to another. As anticipated, cyclic variation in observed vector abundance followed that of the wet and dry seasons. Nevertheless, while in the village of Bana and Souroukoudingan, the vector abundance and associated EIR were highly correlated with seasons, in the village of Pala, the particular local hydrography allowed a low vector density and malaria transmission through the dry season. Additional studies will be needed to have a better understanding of the specific contribution of different malaria vector species in the local malaria transmission. The level of annual malaria transmission remained generally similar from one locality to another (Bana, Pala and Souroukoudingan) and from 1 year to another and remains high despite recent bed net mass-distribution campaigns. Additional vector control tools are urgently needed to complement current malaria control interventions.

## Additional files

**Additional file 1: Table S1.** Monthly catch of the resting anopheline mosquito populations collected indoor (by PSC in 20 houses) within the study villages.

**Additional file 2: Table S2.** Monthly catch of human landing Anopheles *gambiae s.l.* mosquito populations collected indoor and outdoor by HLC (human landing catches) within the study villages.

**Additional file 3: Table S3.** The monthly estimates of *Plasmodium falciparum*’ sporozoite infection rate (SIR) in the studied villages.

## Abbreviations

ANOVA: analysis of variance
CDC: Centers for Disease Control and Prevention
CSP: circumsporozoite protein
df: degree of freedom
DNA: deoxyribonucleic acid
EIR: entomological inoculation rate
ELISA: enzyme linked immunosorbent assay
GLM: generalized linear models
GPS: global positioning system
HLC: human landing catches
HLR: human landing rate
IRSS: Institut de Recherche en Science de la Santé
ITNs: Insecticide-Treated bed Nets
LLINs: long-lasting insecticidal nets
M: million
PCR: polymerase chain reaction
PSC: pyrethroid spray catch
SINE: short interpersed element
SIR: sporozoite infection rate
SIT: sterile insect technique
v/v: volume for volume
