# Supplementary material for: Seasonal malaria vector and transmission dynamics in western Burkina Faso
Source: Malar J. 2019 Apr 2;18:113. doi: 10.1186/s12936-019-2747-5 (PMC6444393; doi:10.1186/s12936-019-2747-5)
Supplement: Supplementary file 1 — Additional file 1: Table S1. Monthly catch of the resting anopheline mosquito populations collected indoor (by PSC in 20 houses) within the study villages. [file 12936_2019_2747_MOESM1_ESM.docx]

**Table S1:** Monthly catch of the resting anopheline mosquito populations collected indoor (by PSC in 20 houses) within the study villages. **A gsl** *=* *Anopheles gambiae s.l*., **OAV** = Other *Anopheles* Vectors (principally *Anopheles nili* and *Anopheles funestus*) and **NVA** = Non-Vector Anopheline mosquitoes. Period is expressed as year/month.

|  | **Bana** | | | | **Souroukoudingan** | | | **Pala** | | | |
| --- | --- | --- | --- | --- | --- | --- | --- | --- | --- | --- | --- |
| **Period** | **A gsl** | **OAV** | | **NVA** | **A gsl** | **OAV** | **NVA** | **A gsl** | **OAV** | | **NVA** |
| 2012/07 | 605 | 1 | 0 | | 202 | 0 | 0 | 191 | 0 | 0 | |
| 2012/08 | 1,672 | 0 | 0 | | 729 | 3 | 0 | 323 | 0 | 2 | |
| 2012/09 | 1,357 | 0 | 0 | | 542 | 1 | 2 | 319 | 0 | 1 | |
| 2012/10 | 1,078 | 0 | 0 | | 609 | 0 | 1 | 392 | 0 | 6 | |
| 2012/11 | 101 | 0 | 0 | | 159 | 0 | 10 | 407 | 1 | 2 | |
| 2012/12 | 39 | 6 | 1 | | 20 | 0 | 0 | 152 | 0 | 0 | |
| 2013/03 | 253 | 2 | 18 | | 81 | 1 | 1 | 91 | 0 | 2 | |
| 2013/04 | 160 | 1 | 2 | | 11 | 0 | 1 | 327 | 1 | 13 | |
| 2013/06 | 148 | 0 | 0 | | 77 | 0 | 3 | 276 | 0 | 1 | |
| 2013/07 | 414 | 0 | 0 | | 316 | 0 | 0 | 489 | 0 | 0 | |
| 2013/08 | 184 | 0 | 0 | | 156 | 0 | 1 | 180 | 0 | 0 | |
| 2013/09 | 1,041 | 2 | 4 | | 366 | 3 | 1 | 745 | 0 | 0 | |
| 2013/10 | 654 | 4 | 2 | | 214 | 4 | 3 | 829 | 1 | 0 | |
| 2013/11 | 60 | 2 | 0 | | 42 | 0 | 0 | 105 | 1 | 0 | |
| 2013/12 | 1 | 0 | 0 | | 1 | 0 | 0 | 6 | 0 | 0 | |
| 2014/01 | 2 | 0 | 0 | | 0 | 0 | 0 | 9 | 0 | 1 | |
| 2014/02 | 11 | 0 | 0 | | 0 | 0 | 1 | 103 | 0 | 5 | |
| 2014/03 | 36 | 0 | 0 | | 15 | 0 | 2 | 94 | 0 | 2 | |
| 2014/04 | 376 | 0 | 1 | | 19 | 0 | 0 | 234 | 0 | 2 | |
| 2014/05 | 176 | 0 | 3 | | 173 | 0 | 0 | 745 | 0 | 3 | |
| 2014/06 | 228 | 0 | 0 | | 117 | 0 | 0 | 87 | 0 | 0 | |
| 2014/07 | 103 | 0 | 0 | | 108 | 0 | 0 | 118 | 0 | 0 | |
| 2014/08 | 440 | 0 | 0 | | 383 | 0 | 0 | 146 | 0 | 0 | |
| 2014/09 | 430 | 0 | 0 | | 588 | 0 | 0 | 342 | 0 | 0 | |
| 2014/10 | 1,182 | 2 | 2 | | 189 | 0 | 14 | 661 | 0 | 0 | |
| 2014/11 | 52 | 2 | 0 | | 39 | 0 | 0 | 159 | 0 | 4 | |
| 2014/12 | 5 | 1 | 0 | | 14 | 0 | 0 | 232 | 0 | 0 | |
| 2015/01 | 3 | 0 | 0 | | 0 | 0 | 0 | 140 | 0 | 0 | |
| 2015/02 | 2 | 0 | 0 | | 1 | 0 | 0 | 282 | 0 | 10 | |
| 2015/03 | 36 | 0 | 0 | | 49 | 0 | 2 | 412 | 0 | 2 | |
| 2015/04 | 201 | 0 | 1 | | 10 | 0 | 0 | 321 | 0 | 1 | |
| 2015/05 | 247 | 0 | 0 | | 23 | 0 | 0 | 319 | 0 | 0 | |
| 2015/06 | 369 | 0 | 0 | | 135 | 0 | 0 | 149 | 0 | 0 | |
| 2015/07 | 318 | 0 | 0 | | 285 | 0 | 0 | 297 | 0 | 0 | |
| 2015/08 | 462 | 0 | 0 | | 677 | 0 | 0 | 121 | 0 | 0 | |
| 2015/09 | 1,499 | 0 | 0 | | 1027 | 0 | 2 | 590 | 0 | 0 | |
| 2015/10 | 510 | 0 | 0 | | 774 | 2 | 20 | 529 | 1 | 1 | |
| 2015/11 | 54 | 1 | 0 | | 71 | 0 | 4 | 267 | 0 | 3 | |
| **Total** | **14,509** | **24** | **34** | | **8,222** | **14** | **68** | **11,189** | **5** | **61** | |
