# Supplementary material for: Seasonal malaria vector and transmission dynamics in western Burkina Faso
Source: Malar J. 2019 Apr 2;18:113. doi: 10.1186/s12936-019-2747-5 (PMC6444393; doi:10.1186/s12936-019-2747-5)
Supplement: Supplementary file 2 — Additional file 2: Table S2. Monthly catch of human landing Anopheles gambiae s.l. mosquito populations collected indoor and outdoor by HLC (human landing catches) within the study villages. [file 12936_2019_2747_MOESM2_ESM.docx]

**Table S2:** Monthly catch of human landing Anopheles *gambiae s.l.* mosquito populations collected indoor and outdoor by HLC (human landing catches) within the study villages.

|  | **Bana** | | | | **Souroukoudingan** | | | **Pala** | | | |
| --- | --- | --- | --- | --- | --- | --- | --- | --- | --- | --- | --- |
| **Period** | **Outdoor** | **Indoor** | | **Total** | **Outdoor** | **Indoor** | **Total** | **Outdoor** | **Indoor** | | **Total** |
| 2012/08 | 1991 | 933 | 2924 | | 727 | 658 | 1385 | 368 | 521 | 889 | |
| 2012/09 | 2768 | 1559 | 4327 | | 1062 | 1169 | 2231 | 474 | 698 | 1172 | |
| 2012/10 | 2781 | 1987 | 4768 | | 196 | 192 | 388 | 158 | 237 | 395 | |
| 2012/11 | 309 | 191 | 500 | | 68 | 78 | 146 | 191 | 313 | 504 | |
| 2012/12 | 19 | 56 | 75 | | 1 | 6 | 7 | 66 | 91 | 157 | |
| 2013/03 | 53 | 71 | 124 | | 1 | 0 | 1 | 29 | 27 | 56 | |
| 2013/04 | 164 | 202 | 366 | | 0 | 0 | 0 | 83 | 129 | 212 | |
| 2013/06 | 152 | 104 | 256 | | 4 | 5 | 9 | 67 | 270 | 337 | |
| 2013/07 | 1363 | 1328 | 2691 | | 42 | 68 | 110 | 225 | 409 | 634 | |
| 2013/08 | 888 | 775 | 1663 | | 536 | 747 | 1283 | 632 | 903 | 1535 | |
| 2013/09 | 3351 | 3461 | 6812 | | 1333 | 1932 | 3265 | 577 | 1046 | 1623 | |
| 2013/10 | 2563 | 2161 | 4724 | | 225 | 324 | 549 | 441 | 580 | 1021 | |
| 2013/11 | 112 | 122 | 234 | | 15 | 39 | 54 | 61 | 68 | 129 | |
| 2013/12 | 2 | 3 | 5 | | 1 | 1 | 2 | 2 | 24 | 26 | |
| 2014/01 | 2 | 5 | 7 | | 0 | 0 | 0 | 14 | 31 | 45 | |
| 2014/02 | 7 | 2 | 9 | | 0 | 2 | 2 | 44 | 58 | 102 | |
| 2014/03 | 11 | 12 | 23 | | 0 | 0 | 0 | 24 | 44 | 68 | |
| 2014/04 | 144 | 131 | 275 | | 0 | 2 | 2 | 52 | 87 | 139 | |
| 2014/05 | 198 | 146 | 344 | | 33 | 71 | 104 | 463 | 472 | 935 | |
| 2014/06 | 380 | 584 | 964 | | 58 | 171 | 229 | 174 | 244 | 418 | |
| 2014/07 | 295 | 275 | 570 | | 74 | 209 | 283 | 353 | 489 | 842 | |
| 2014/08 | 699 | 746 | 1445 | | 461 | 836 | 1297 | 349 | 358 | 707 | |
| 2014/09 | 3171 | 2242 | 5413 | | 1682 | 1794 | 3476 | 975 | 920 | 1895 | |
| 2014/10 | 1634 | 1409 | 3043 | | 260 | 349 | 609 | 532 | 407 | 939 | |
| 2014/11 | 43 | 50 | 93 | | 17 | 12 | 29 | 189 | 168 | 357 | |
| 2014/12 | 0 | 4 | 4 | | 1 | 0 | 1 | 250 | 475 | 725 | |
| 2015/01 | 1 | 1 | 2 | | 0 | 0 | 0 | 22 | 47 | 69 | |
| 2015/02 | 0 | 0 | 0 | | 0 | 0 | 0 | 173 | 120 | 293 | |
| 2015/03 | 13 | 34 | 47 | | 4 | 1 | 5 | 131 | 152 | 283 | |
| 2015/04 | 85 | 85 | 170 | | 0 | 1 | 1 | 118 | 75 | 193 | |
| 2015/05 | 76 | 148 | 224 | | 1 | 1 | 2 | 126 | 127 | 253 | |
| 2015/06 | 296 | 512 | 808 | | 29 | 55 | 84 | 152 | 223 | 375 | |
| 2015/07 | 1047 | 1599 | 2646 | | 146 | 330 | 476 | 180 | 310 | 490 | |
| 2015/08 | 1432 | 1769 | 3201 | | 1575 | 1654 | 3229 | 612 | 707 | 1319 | |
| 2015/09 | NA | NA | NA | | NA | NA | NA | NA | NA | NA | |
| 2015/10 | 3286 | 3125 | 6411 | | 652 | 711 | 1363 | 1313 | 1964 | 3277 | |
| 2015/11 | 112 | 142 | 254 | | 13 | 66 | 79 | 810 | 711 | 1521 | |
| **Total** | **29,448** | **25,974** | **55,422** | | **9,217** | **11,484** | **20,701** | **10,430** | **13,505** | **23,935** | |

The monthly results are the total mosquito caught from 4 different houses during 4 consecutive nights. Period is expressed as year/month.
