# Supplementary material for: Seasonal malaria vector and transmission dynamics in western Burkina Faso
Source: Malar J. 2019 Apr 2;18:113. doi: 10.1186/s12936-019-2747-5 (PMC6444393; doi:10.1186/s12936-019-2747-5)
Supplement: Supplementary file 3 — Additional file 3: Table S3. The monthly estimates of Plasmodium falciparum’ sporozoite infection rate (SIR) in the studied villages. [file 12936_2019_2747_MOESM3_ESM.docx]

**Table S3:** The monthly estimates of *Plasmodium falciparum*’ sporozoite infection rate (SIR) in the studied villages.

| Period | Bana | Souroukoudingan | Pala |
| --- | --- | --- | --- |
|  | SIR (n ; N) | SIR (n ; N) | SIR (n ; N) |
| 2012/08 | 0.050 (3 ; 60) | 0.017 (1 ; 60) | NA |
| 2012/09 | 0.000 (0 ; 60) | 0.017 (1 ; 60) | 0.050 (3 ; 60) |
| 2012/10 | 0.100 (6 ; 60) | 0.100 (6 ; 60) | 0.117 (7 ; 60) |
| 2012/11 | 0.033 (2 ; 60) | 0.100 (6 ; 60) | 0.070 (4 ; 57) |
| 2012/12 | 0.022 (1 ; 46) | 0.000 (0 ; 07) | 0.160 (8 ; 50) |
| 2013/03 | 0.000 (0 ; 21) | NA | 0.000 (0 ; 24) |
| 2013/04 | 0.000 (0 ; 60) | 0.000 (0 ; 03) | 0.000 (0 ; 03) |
| 2013/06 | 0.050 (3 ; 60) | 0.167 (1 ; 06) | 0.017 (1 ; 60) |
| 2013/07 | 0.017 (1 ; 60) | 0.196 (10 ; 51) | 0.167 (10 ; 60) |
| 2013/08 | 0.000 (0 ; 60) | 0.067 (4 ; 60) | 0.089 (5 ; 56) |
| 2013/09 | 0.000 (0 ; 60) | 0.019 (1 ; 53) | NA |
| 2013/10 | 0.033 (2 ; 60) | 0.050 (3 ; 60) | NA |
| 2013/11 | 0.017 (1 ; 60) | 0.000 (0 ; 34) | 0.067 (4 ; 60) |
| 2013/12 | 0.167 (1 ; 06) | 0.500 (1 ; 02) | 0.192 (5 ; 26) |
| 2014/01 | 0.000 (0 ; 06) | NA | 0.000 (0 ; 37) |
| 2014/02 | 0.000 (0 ; 11) | NA | 0.000 (0 ; 106) |
| 2014/03 | 0.000 (0 ; 21) | 0.020 (1 ; 50) | 0.000 (0 ; 59) |
| 2014/04 | 0.083 (5 ; 60) | NA | 0.000 (0 ; 52) |
| 2014/05 | 0.000 (0 ; 60) | NA | 0.000 (0 ; 60) |
| 2014/06 | 0.050 (3 ; 60) | 0.000 (0 ; 60) | 0.000 (0 ; 58) |
| 2014/07 | 0.200 (12 ; 60) | 0.086 (5 ; 58) | 0.000 (0 ; 60) |
| 2014/08 | 0.017 (1 ; 60) | 0.050 (3 ; 60) | 0.017 (1 ; 60) |
| 2014/09 | 0.000 (0 ; 60) | 0.000 (0 ; 60) | 0.000 (0 ; 60) |
| 2014/10 | 0.000 (0 ; 60) | 0.034 (2 ; 59) | 0.067 (4 ; 60) |
| 2014/11 | 0.048 (2 ; 42) | 0.000 (0 ; 31) | 0.083 (5 ; 60) |
| 2014/12 | 0.000 (0 ; 02) | NA | 0.033 (2 ; 60) |
| 2015/03 | 0.000 (0 ; 33) | 0.000 (0 ; 06) | 0.000 (0 ; 60) |
| 2015/04 | 0.000 (0 ; 58) | NA | 0.000 (0 ; 60) |
| 2015/05 | 0.018 (1 ; 57) | NA | 0.000 (0 ; 60) |
| 2015/06 | 0.033 (2 ; 60) | 0.033 (2 ; 60) | 0.000 (0 ; 60) |

The period is expressed as year/month. The numbers in bracket are respectively “n”: the number of infected mosquitoes found (positive to ELISA test) and “*N*”: the total number of mosquitoes processed during the ELISA-CSP analysis in the corresponding month and village. “SIR” is the Sporozoite Infection Rate and “NA” means that no mosquitoes were available for processing in the corresponding months.
